# Multiscale modeling of genome organization with maximum entropy optimization

**DOI:** 10.1063/5.0044150

**Published:** 2021-07-01

**Authors:** Xingcheng Lin, Yifeng Qi, Andrew P. Latham, Bin Zhang

**Affiliations:** Department of Chemistry, Massachusetts Institute of Technology, Cambridge, Massachusetts 02139, USA

## Abstract

Three-dimensional (3D) organization of the human genome plays an essential role in all DNA-templated processes, including gene transcription, gene regulation, and DNA replication. Computational modeling can be an effective way of building high-resolution genome structures and improving our understanding of these molecular processes. However, it faces significant challenges as the human genome consists of over 6 × 10^9^ base pairs, a system size that exceeds the capacity of traditional modeling approaches. In this perspective, we review the progress that has been made in modeling the human genome. Coarse-grained models parameterized to reproduce experimental data via the maximum entropy optimization algorithm serve as effective means to study genome organization at various length scales. They have provided insight into the principles of whole-genome organization and enabled *de novo* predictions of chromosome structures from epigenetic modifications. Applications of these models at a near-atomistic resolution further revealed physicochemical interactions that drive the phase separation of disordered proteins and dictate chromatin stability *in situ*. We conclude with an outlook on the opportunities and challenges in studying chromosome dynamics.

## I. INTRODUCTION

The genome is often hailed as the blueprint of life. It instructs the development and operation of an entire organism. Advancement in sequencing techniques has made possible the determination of nucleic acid sequences that make up the genome for many species, including the completion of the human genome project.^1,2^ These sequences provide valuable information about the genome’s function and a glimpse into the meaning of life at the atomic level.^3^ They allowed discovering and cataloging coding regions or genes that produce protein molecules and define the distinctive features of individual cells. Notably, genes only make up a small fraction of the genome. While the rest of the genome is not transcribed into proteins, it helps encode cellular diversity found in multicellular organisms by regulating the amount of proteins genes produced.^4^

**FIG. 1.**
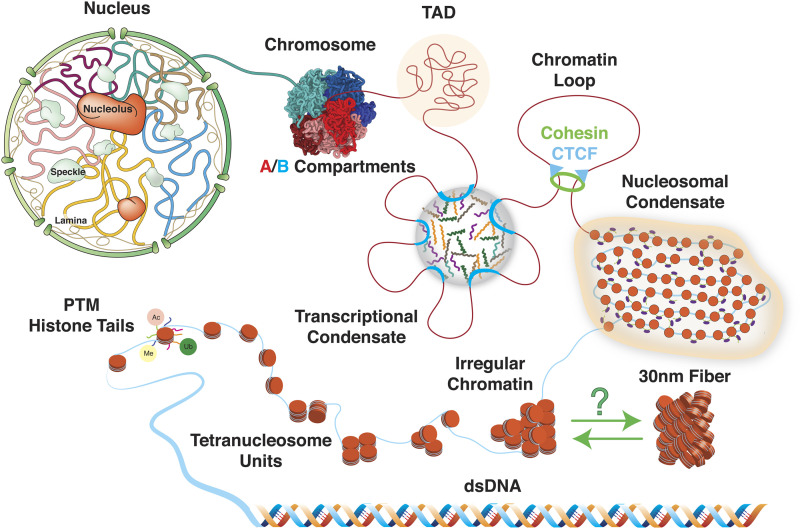
Illustration of the many layers of three-dimensional genome organization. In eukaryotes, the double-stranded DNA first wraps around histone proteins (orange) to form nucleosomes. The N-terminal tails of histone proteins are subject to a wide range of post-translational modifications (PTM), including acetylation (Ac), methylation (Me), and ubiquitination (Ub). A string of nucleosomes, or chromatin, may compact into irregular structures and nucleosomal condensates, although regular structures have also been seen *in vitro*. At larger scales, genomic segments that are far apart in sequence can come in contact due to the formation of chromatin loops, transcriptional condensates, and topologically associating domains (TAD). TADs of similar properties may phase separate, resulting in the compartmentalization of chromosomes into regions enriched with heterochromatin (*B* compartment, blue) or euchromatin (*A* compartment, red). These two chromatin types differ in their compactness, gene density, and nuclear localization. Inside the nucleus, individual chromosomes often occupy non-overlapping regions to form territories.

One of the means the non-coding regions regulate gene expression is through three-dimensional (3D) genome organization. The genome, much like enzymes, must fold in 3D to form active sites that catalyze the progression of chemical reactions.^5–7^ As shown in Fig. 1, the first layer of eukaryotic genome organization is the formation of nucleosomes by wrapping DNA around histone proteins. Access to nucleosomal DNA is restricted due to its tight binding with histones.^8^ Therefore, regulating nucleosome conformation serves as an effective strategy for controlling genome accessibility and modulating gene expression levels.^9,10^ Similar arguments can be made for a string of nucleosomes, i.e., chromatin, as compacting chromatin by stacking nucleosomes close to each other will again limit DNA accessibility and exclude the binding of the transcriptional machinery.^11,12^ At even larger scales, genome folding could bring regulatory elements (enhancers and promoters) that are far apart in the linear sequence (10kb to 1Mb) to spatial proximity.^13^ Such contacts could achieve targeted control of gene expression by selectively localizing the transcriptional machinery to cell-type-specific genes. A detailed characterization of the genome organization and its dependence on the non-coding regions could improve our understanding of gene regulation in eukaryotes.

Many questions regarding what the genome structure is and how the genome folds remain to be addressed.^14^ Globally, the genome organization appears to be poised on the border between order and disorder. While many studies have uncovered non-random structural features, the genome is largely amorphous and does not adopt a single unique conformation.^15^ How specific interactions emerge from the large pool of non-specific contacts is mostly unknown.^16^ It is tempting to assume that the genome adopts a similar sequence to structure relationship as that has been widely accepted for proteins. If so, what exactly is the sequence that dictates the genome organization? The answer to this question is not apparent and cannot be simply the sequence of nucleotides. Epigenetic modifications^17–19^ may be the key to forming distinct genome organizations to encode unique gene expression profiles in cells that share identical DNA sequences. Equally important questions arise at smaller scales on the level of single genes (∼10kb). While a seemingly more straightforward problem, the structure for a string of nucleosomes remains controversial.^20–22^ There is an ongoing debate regarding whether chromatin *in situ* adopts the same set of conformations as those seen *in vitro*. Its interaction with protein molecules inside the nucleus may drive chromatin into phase-separated condensates,^23,24^ which renders a high-resolution structural characterization challenging due to its dynamic nature. Addressing these questions will improve our understanding of gene regulatory mechanisms and is promising for uncovering novel genome engineering approaches to alter the genome organization for more targeted and reversible therapeutic approaches. We hope this perspective may introduce the chemical physics community to the fascinating genome folding problem and inspire more theoretical and physical chemistry research efforts.

Much like its success in studying protein folding, computational modeling could be powerful tools for interpreting experimental data, exploring mechanistic hypotheses, and predicting genome organization *de novo*.^25–27^ Many simulation tools introduced for protein folding can indeed be transferred to study genome organization. Computational modeling will face unique challenges not seen in protein folding as well, presenting opportunities for the development of new methods and theories. *First*, the human genome consists of over 6 × 10^9^ base pairs, a system size that exceeds the capacity of all-atom explicit solvent simulations. Even a small chromatin segment, when fully solvated, can lead to systems of several millions of atoms in size. For whole-genome modeling, coarse-graining will be necessary. *Second*, while the physicochemical interactions that stabilize protein structures are relatively well understood, the same cannot be said for the genome. In particular, many long-range contacts between genomic segments are mediated by protein molecules, the identity of which has yet to be revealed. Since the system’s exact composition is lacking, multi-scale approaches^28,29^ that aim at deriving coarse-grained force fields from atomistic simulations are inapplicable for parameterizing whole-genome models. Further development of the coarse-grained modeling strategy is needed. *Finally*, the genome is inherently a non-equilibrium system, and its conformational ensemble, in principle, cannot be described with equilibrium statistical mechanics.^30^ Efficient algorithms are needed to simulate the impact of ATP-driven enzymes that consume energy to break the detailed balance, so are theoretical tools for interpreting simulation results.

In this perspective, we will review the progress in modeling the human genome and outline the challenges that lie ahead. It is organized as follows: We first discuss modeling efforts at the entire chromosome and the whole-genome level. Particular focus will be devoted to the data-driven mechanistic modeling approach that is effective at unraveling the principles of genome organization and enabling *de novo* structural prediction. In Sec. III, we transition to studies on a much smaller scale for a string of nucleosomes. Arguments will be made to near-atomistic models that can potentially bridge the gap between mesoscopic models and atomistic simulations to characterize chromatin organization *in situ*. While the modeling strategies in Secs. II and III differ significantly, maximum entropy optimization serves as an effective strategy for parameterizing coarse-grained models in both cases. We acknowledge that chromatin organization between the two scales considered here is of critical importance as well, and see the excellent reviews on such topics.^31–33^ Finally, we provide an outlook on the opportunities and challenges in modeling chromosome dynamics.

## II. STRUCTURE FOR THE ENTIRE GENOME

The human genome consists of over 6 × 10^9^ base pairs, and each one of the 46 chromosomes accounts for tens or hundreds of million bases (MB) in length. When fully extended, the genome accounts for ∼2 m. Formation of nucleosomes that are 10 nm in size and 200 bp in sequence length reduces the genome length by almost an order of magnitude to 0.3 m, a number that still vastly exceeds the size of a typical cell nucleus (∼10 *μ*m in diameter). Therefore, additional folding and compaction must occur beyond nucleosomes to fit the genome inside the nucleus. We note that most probable polymer configurations are not straight lines but resemble random coils with size *R*_*g*_ ∝ *N*^1/2^*a*,^34^ where *N* is the number of nucleosomes and *a* is the nucleosome diameter. For a naive estimation with all chromosomes connected together, we have *N* ≈ 3 × 10^7^, *a* = 10 nm, and *R*_*g*_ ≈ 55 *µ*m. Further collapsing coiled configurations into globules that scale as *N*^1/3^*a* ≈ 3 *µ*m does allow the genome to fit inside the nucleus. Great progress has been made toward understanding how the folding proceeds and what the final folded configuration is. Many research groups have contributed to addressing this inherently multi-scale problem using a wide range of tools.

### A. Experimental characterization of genome organization

The genome is large and amorphous, and the nucleus environment is heterogeneous and crowded. These features render a high-resolution structural characterization of genome organization challenging. Many well-established techniques that succeed at producing atomic structures for proteins, including x-ray crystallography and Nuclear Magnetic Resonance (NMR), are not directly applicable to the genome. Instead, structural information of the genome was often derived from electron microscopy (EM) or fluorescence *in situ* hybridization (FISH). Applications of EM imaging have led to the discovery of different chromatin types, including heterochromatin and euchromatin that differ in their degree of compactness, gene density, and nuclear localization.^35,36^ Electron micrographs of metaphase chromosomes swollen by divalent ions further revealed the presence of radial loops.^37^ Meanwhile, FISH, which uses a probe DNA sequence attached with a fluorophore to hybridize with a corresponding chromosome region via base pairing, allows direct visualization of specific genomic loci with fluorescence microscopy. Experiments that employ multiple probes to measure the spatial distance between genomic loci support the presence of looped structures in interphase as well.^38–40^ Labeling whole chromosomes further revealed that they tend to demix and occupy non-overlapping spatial regions termed chromosome territories.^41–43^ While imaging-based techniques have successfully characterized chromosome morphology and large scale organization, traditionally, they have not been effective at studying fine-scale structures due to the limited resolution.

**FIG. 2.**
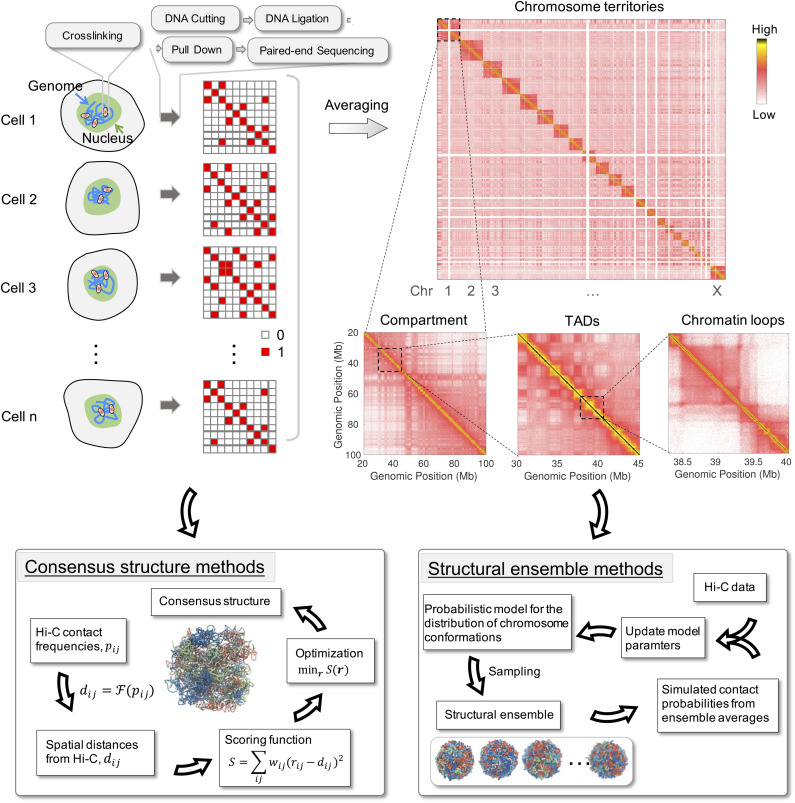
Computational modeling of genome organization with Hi-C data. Top: illustration of the experimental protocol used in population Hi-C experiments (see the text for details). An example contact probability map for the genome from GM12878 cells is shown on the right, with the probability decreasing from yellow to red and to white. Bottom: illustration of the two popular methods used in building genome structures from Hi-C data. In consensus structure methods (left), pairwise Hi-C contact frequencies (*p*_*ij*_) are first transformed to distances (*d*_*ij*_) via a mapping function F. These distances can be used as constraints to refine computer models and derive consensus structures for the genome. An ensemble of structures can also be used (right) to reproduce Hi-C contact frequencies without converting them to distances. These structural ensemble methods often describe the structures with a probabilistic model and use iterative algorithms to update model parameters.

High-throughput sequencing-based techniques, including genome-wide chromosome conformation capture (Hi-C) and related methods,^44–46^ have served as effective alternatives for studying genome organization. As shown in Fig. 2, Hi-C experiments start by cross-linking DNA segments in spatial proximity inside the nucleus using formaldehyde. The genome is then fragmented with restriction enzymes to result in pairs of contacting segments. Sequencing these contacting pairs reveals their identity, which can be mapped to the reference genome to determine the genomic position of individual loci. For a single structure, this protocol produces a binary contact matrix, each entry of which being either 0 or 1 to indicate whether the corresponding genomic pair is in contact or not. When averaged over a population of millions of cells, as often done in experiments, the protocol produces a contact frequency, or probability, map. The contact map provides rich information on the arrangement of the genome inside the nucleus. Its resolution is bound by the frequency at which restriction enzymes cut the DNA. Since the average spacing between sequences recognized by restriction enzymes is ∼1kb, Hi-C experiments can, in principle, characterize genome organization at high resolution.

Hi-C experiments have confirmed findings from imaging studies and revealed many previously unknown features of genome organization.^47–49^ A typical contact probability map from GM12878 cells is shown in Fig. 2, where the probability decreases from yellow to red and to white. Consistent with the formation of chromosome territories, individual chromosomes appear as squares of high contact frequency along the diagonal. Zooming into individual chromosomes revealed block-wise checkerboard patterns that indicate the presence of two chromatin types, often termed *A*/*B* compartments^45^ and correlated with euchromatin and heterochromatin. The yellow squares appearing along the diagonal line support the formation of topologically associating domains (TADs) with enriched contacts inside the domain than contacts across domains.^50–52^ A subset of the TADs exhibits strong contact signals at the corner, supporting the formation of looped structures with pronounced interactions localizing at the ends.^51^

We note that Hi-C experiments are not without limitations. In particular, averaging over a population of cells is often necessary to achieve improved statistics but also casts doubt on the relevance of observed structural motifs in individual cells.^53–55^ Hi-C experiments also do not directly measure 3D distances. Sometimes, a higher contact frequency does not translate into a shorter distance,^56,57^ rendering a structural interpretation of the contact map less straightforward. The recent development of super-resolution imaging^58–61^ and *in situ* genome sequencing^62^ techniques can potentially overcome the shortcomings of traditional imaging and Hi-C to provide high-resolution single-cell structural models for the genome.^43,63^

### B. Computational modeling of genome organization

Computational modeling has been valuable for studying genome organization. In addition to providing intuitive 3D structural views of the genome, computational approaches are effective at falsifying hypotheses and identifying the ones that are consistent with experimental data.^26,27,64,65^ Due to the complexity of the genome organization and our limited knowledge of the folding mechanism, various modeling strategies have been employed in prior studies. We classify them as data-driven approaches that mainly rely on experimental data for structural modeling and hypothesis-driven approaches in which experimental data mostly serve for validation purposes.

#### 1. Data-driven modeling approaches

##### a. Consensus structure refinement approaches.

Hi-C experiments measure the contact probabilities between genomic loci. Assuming a direct mapping between the probabilities and spatial distances exists, one can convert Hi-C data into a distance matrix. Consensus structures can be derived from this matrix by minimizing scoring functions that measure the difference between simulated and experimental distances (Fig. 2). We note that similar ideas have been widely used in predicting protein structures^66^ in which the distances between amino acids can be derived from NMR measurements.

The scoring functions are often complex and are of high dimension. Numerous algorithms have been introduced for their efficient optimization to study genome organization in different organisms.^67–78^ Duan *et al.* constructed a three-dimensional model of the *Saccharomyces cerevisiae* genome using the interior-point gradient-based methods.^67^ The model effectively captures intra- and inter-chromosomal contacts and reveals notable structural features, including individual chromosomal folding and centromere-anchored inter-chromosomal interactions. Molecular dynamics simulations,^73^ Monte Carlo sampling,^76^ simulated annealing,^69,70,75^ Bayesian inference,^77^ and manifold embedding^78–80^ have also been used for structure building. These techniques allow for the construction of multiple models consistent with experimental data and have provided insight into the connection between 3D structures and 1D genomic features. Notably, Yildirim and Feig^76^ derived structural models for *Caulobacter crescentus* at the single base-pair resolution, thanks to its small genome size. The Mozziconacci laboratory introduced a 3D shortest-path reconstruction method based on the multidimensional scaling-based algorithm.^68^ This method is computationally efficient and can produce robust results for sparse and noisy contact maps. Algorithms that separate out intra- and inter-chromosomal contacts for high-resolution structural reconstruction at low computational costs have also been developed.^74^ Besides the applications on population Hi-C data, distance-based methods have recently been generalized to single-cell Hi-C^81–84^ and ChIA-PET data.^71,72^

##### b. Structural ensemble refinement approaches.

The pursuit of a consensus structure, while desirable for simplified interpretations, can mask the intrinsic heterogeneity of genome organization within a cell population. Since population Hi-C experiments report the contact frequencies averaged over many cells, there will likely be an ensemble of structures that collectively reproduce the data.^53,81^

To account for large conformational fluctuations around the mean structure, Sasai and co-workers designed a modeling approach using statistical potentials centered at the distances converted from contact probabilities.^85,86^ When applying this approach to the yeast genome, the authors explained experimental chromosome distributions and uncovered a correlation between the transcriptional level of genes and their spatial distribution.

Numerous groups have developed computational techniques to directly fit Hi-C data with an ensemble of structures and avoid converting experimental contact frequencies to spatial distances, the functional form for which remains unknown.^87–95^ For example, Alber and co-workers introduced a probabilistic framework based on the maximum likelihood optimization algorithm to reproduce Hi-C contacts with averages from a population of thousands of genome structures.^87–89^ Applications of this algorithm revealed the presence of centromere clusters in GM12878 cells and the role of these clusters in positioning chromosomes in the nucleus. Liang and co-workers introduced CHROMATIX, a method that first identifies a minimal set of sufficient interactions and deconvolutes these interactions into a population of single-cell contact states that can then be used for structure reconstruction.^90–93^ This method succeeds in characterizing higher-order interactions between multiple genomic regions and structural changes during embryogenesis in Drosophila.

Zhang and Wolynes took a more statistical mechanical approach by transforming the problem of structural refinement into parameterization of pairwise interactions between genomic loci, i.e., force field optimization.^94,96^ In particular, assuming that the genome organization from a population of cells can be approximated with the Boltzmann distribution of an energy function, finding the set of structures that reproduce experimental data is equivalent to determining the corresponding energy function. Using experimental data to parameterize or improve force field is indeed a topic well studied in the protein folding community. Low-resolution data, including small-angle x-ray scattering (SAXS), Förster resonance energy transfer (FRET), and nuclear magnetic resonance (NMR), are frequently used to improve the determination of protein structures.^97–99^ While many functional forms can be defined for the energy function, the one that introduces the least bias, i.e., the optimal model, is derived by maximizing the excess entropy based on the information theory.^100^

The starting point of the Zhang and Wolynes approach is a beads-on-a-string model to mimic the continuity of the DNA molecule, where each bead represents a genomic segment of fixed length. A corresponding energy function, $U\left(r\right)$, which usually contains terms that account for bonding, bending, and excluding volume effect, can be defined to evaluate the stability of polymer configurations. Additional energy terms that mimic the confinement effect of neighboring chromosomes and the nuclear envelope can also be included. The equilibrium Boltzmann distribution is

$$P\left(r\right)=\frac{e^{-\beta U\left(r\right)}}{\int e^{-\beta U\left(r\right)}dr},$$ (1)

where *β* = 1/*k*_*B*_*T*. Defining a threshold function *f*(*r*_*ij*_; *r*_*o*_) that switches from 0 to 1 as the distance *r*_*ij*_ decreases below *r*_*o*_, one can compute the contact probability for a pair of genomic loci as $p_{ij}^{\text{sim}}=\int f\left(r_{ij}\right)P\left(r\right)dr$. *r*_*o*_ can be viewed as the minimal contact distance necessary for cross-linking. Since *U*(***r***) does not capture any biological interactions that chromosomes experience inside the nucleus, the simulated contact probabilities are not expected to reproduce Hi-C data.

To improve the model’s biological relevance, correction terms can be introduced to the energy function. Maximum entropy optimization has become widely popular in recent years^99,101–103^ as an efficient means to improve the agreement between modeling and experiment. Under the maximum entropy framework, one seeks a new model, *U*_ME_(***r***), and a corresponding probability distribution function $P_{\text{ME}}\left(r\right)=e^{-\beta U_{\text{ME}}\left(r\right)}/\int e^{-\beta U_{\text{ME}}\left(r\right)}dr$. Importantly, for the new model to reproduce experimental contact probabilities, i.e., $\int f\left(r_{ij}\right)P_{\text{ME}}\left(r\right)dr\equiv p_{ij}^{\exp}$, while maximizing the excess entropy, −∫*P*_ME_(***r***)ln[*P*_ME_(***r***)/*P*(***r***)], its energy function has a unique solution defined as

$$U_{\text{ME}}\left(r\right)=U\left(r\right)+\underset{i>j}{\sum }\alpha_{ij}f\left(r_{ij}\right),$$ (2)

where *α*_*ij*_ are Lagrangian multipliers, the value of which ensures the agreement between simulation and experiment. They can be determined via an efficient iterative optimization procedure, as detailed in Ref. 94. We refer the interested readers to Refs. 102 and 104–106 for detailed reviews on maximum entropy optimization.

From the energy function, an ensemble of chromosome structures can be constructed via molecular dynamics simulations. Notably, the structural ensemble is guaranteed to reproduce experimental contact probabilities. By inspecting the simulated structures with knot invariants, Zhang and Wolynes found that interphase chromosomes are free of topological entanglements. Their result generalizes beyond using the scaling exponent of contact probability as a function of sequence separation to support knot-free genome organization.^45,107^ Since the maximum entropy model matches the complete set of experimental data with minimal assumptions of genome organization, it produces the least biased or “most likely” chromosome structures based on the given knowledge. Conclusions drawn from these structures are thus more reliable than those based on specific features of Hi-C data. When applied to mitotic chromosomes, the maximum entropy approach further revealed cylindrical conformations with a helical twisting along the central axis. This twisting was later confirmed in experiments with higher resolution Hi-C data.^108^ Chu and Wang further studied the conformational transition between interphase and metaphase chromosomes and revealed a two-stage pathway for chromosome compaction.^109^

Tiana and co-workers applied a similar maximum entropy optimization approach to derive pairwise contact energies from Hi-C. Monte Carlo sampling of the resulting energy function predicted chromatin conformation heterogeneity within a single TAD and provided insight into asymmetric expression of *X*-chromosome inactivation.^110,111^ Shi and Thirumalai further applied the maximum entropy approach to recreating the structural ensemble by reproducing the pairwise mean distances converted from Hi-C probability.^112^ They characterized the heterogeneity of chromosome structures within the same cell type and across different types.

#### 2. Hypothesis-driven modeling approaches

The data-driven modeling approaches are useful for reconstructing chromosome structures. However, they face difficulty at addressing questions regarding why chromosomes adopt specific conformations. Hypothesis-driven approaches are more suited for exploring genome organization mechanisms and have also been widely used in parallel.

**FIG. 3.**
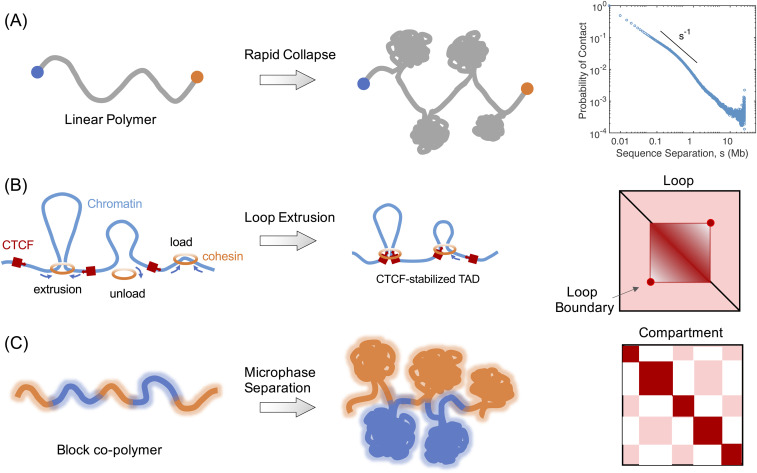
Illustration of the various mechanisms proposed for genome folding. (a) The metastable fractal globule forms in a process that drives the rapid collapse of a long polymer from an expanded, knotless configuration. The two ends do not have enough time to take part in the collapse, and the polymer remains knotless. (b) In the extrusion model, chromatin loops form as a result of the processive movement of Cohesin molecules along the DNA. CTCF molecules act as blockers to stop Cohesin extrusion, explaining the accumulation of the two proteins at loop boundaries. (c) Microphase separation of block copolymers can lead to contact patterns similar to the compartmentalization seen in Hi-C maps.

Given that chromosomes are long macromolecules, whose equilibrium conformations will become entangled under confinement, Grosberg *et al.* proposed the fractal globule as a model for chromosome organization^113^ [see Fig. 3(a)]. Conformations of the fractal globule are free of entanglements, the presence of which may hinder gene transcription and chromosome separation during mitosis. The fractal globule corresponds to a metastable state that forms when the polymer is rapidly collapsed from an extended conformation. It exhibits a different scaling law from equilibrium conformations regarding the decay of the contact probability as a function of sequence separation. Remarkably, early Hi-C experiments indeed appear to support the scaling exponent predicted by the fractal globule.^45,107^ Metastable conformations can persist over the lifetime of a cell, as shown by Rosa and Everaers,^114^ due to the slow relaxation time of polymer topology in a crowded environment.

It is worth mentioning that when interpreted strictly, as pointed out by Huang *et al.*,^115^ the fractal globule is probably too ideal of a model for genome organization. Its self-similarity property conflicts with the spatial heterogeneity of chromatin packing. Huang *et al.* further introduced a self-returning random walk (SRRW) model that achieves simultaneous high self-interacting frequency and high space-filling heterogeneity. This model makes several predictions consistent with experimental observations, including highly porous chromatin structures, flexible higher-order folding, and irregularly shaped chromosome territory.

In addition to their global topological organization, specific structural features of chromosomes have also been studied extensively. For example, loops have received significant attention in early models of interphase chromosomes to explain the scaling behavior of spatial distances as a function of genomic separation measured by FISH experiments.^38,39,116,117^ High-resolution Hi-C data provided direct evidence for the presence of loops in interphase chromosomes.^51^ Importantly, connecting the contact map with the underlying DNA sequence further allowed proposing and validating molecular mechanisms of loop formation. For example, the extrusion model^118,119^ assumes that these loops form by the processive movement of cohesin molecules that bring close genomic segments far apart in sequence until stopped by CTCF molecules [see Fig. 3(b)]. This model explains the enriched contact within a TAD and the flanking of TADs and loop boundaries with Cohesin and CTCF molecules. Several predictions of the extrusion model have been validated with perturbative Hi-C^120–124^ and *in vitro* experiments.^125,126^ Other groups have also highlighted the importance of protein binding in chromosome organization.^127,128^ In these models, the strong and specific interactions between protein and chromatin drive the contacts within a TAD and the separation between neighboring TADs.

At a larger scale, both imaging experiments and the checkerboard patterns in Hi-C contact maps support the compartmentalization of active and inactive chromatin. Compartmentalization can arise from the microphase separation seen in block copolymers.^129–135^ Block copolymers are reasonable models for chromosomes since different chromatin segments can exhibit different chemical properties due to their unique histone modification^136^ and protein association^137,138^ patterns. Additionally, non-equilibrium processes, such as transcription and chromatin remodeling, could contribute to the microphase separation as well. In a simplified model, these processes could give rise to higher effective temperature for active chromatin,^139,140^ and the hotter chromatin will separate from the cooler one as a result of their difference in mobility.^141,142^ Finally, Wang *et al.* treated the 3D chromosome arrangement as an ellipsoid packing problem to understand the correlation between chromosome positioning and cell geometry.^143^

As is evident from the above discussions, multiple models and mechanisms often explain chromosome organization at various scales and reproduce specific features of the experimental data equally well. It is plausible that numerous players co-exist to organize the genome. Quantitatively evaluating the relative significance of various mechanisms is challenging, however, due to the presence of free model parameters whose values are not known *a priori*.

### C. Perspective: Data-driven mechanistic modeling

Coupling the data- and hypothesis-driven approaches together can potentially produce a powerful strategy for modeling genome organization. This strategy ensures the biological relevance of simulated genome structures since all model parameters will be derived from Hi-C data. In the meantime, it will be well suited for mechanistic investigation as the polymer model’s energy function will be designed explicitly from biological factors that are known to contribute to genome organization. Pioneering work by Di Pierro *et al.* has shown that a phase separation model among sub-compartments succeeds at reproducing Hi-C data and recapitulating many known aspects of chromosome organization.^134^

#### 1. Exploring the principles of genome organization

**FIG. 4.**
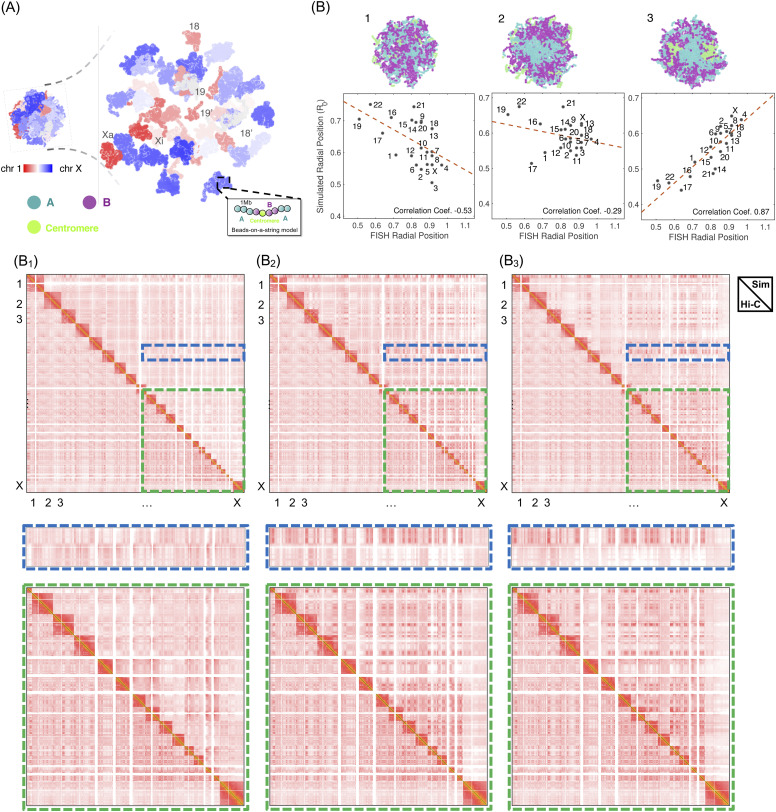
Data-driven mechanistic modeling of the whole-genome organization. (a) An example configuration of the diploid human genome colored from red to white and to blue with increasing chromosome ID. Each chromosome is modeled as a string of beads that can either be *A* (cyan) compartments, *B* (purple) compartments, or centromeres (green). (b) Example genome configurations colored by bead types (top), comparison between simulated and experimental chromosome radial positions (bottom), and comparison between simulated (upper triangle) and experimental (lower triangle) genome-wide contact maps for three genome models. In model 1, only one set of parameters was used to model intra- and inter-chromosomal interactions, while two sets of independent parameters were used in model 2. In model 3, in addition to the use of independent parameters for intra- and inter-chromosomal interactions, the centromeric regions were explicitly represented with a new type.

We followed the data-driven mechanistic modeling strategy and introduced a polymer model to study the 3D organization of the human diploid genome at 1Mb resolution [Fig. 4(a)].^144^ One particular hypothesis that we aimed to evaluate is whether the whole-genome organization can be understood from phase separation of different chromatin types. Toward that end, we designed a block copolymer model that includes 46 polymers in spherical confinement. Each polymer represents a chromosome whose length is determined by the underlying DNA sequence. The spherical confinement mimics the effect of the nuclear envelope. A bead in the polymer was either labeled *A* or *B* to represent the two compartment types seen in Hi-C. The compartment profiles for individual chromosomes were determined from eigenanalysis on the correlation matrix of intra-chromosomal Hi-C contact maps.^45^ For this polymer model, the energy function is defined as follows:

$$\begin{array}{ll} U_{\text{Genome}}\left(r\right) & =\underset{I}{\sum }U\left(r_{I}\right)+\underset{I}{\sum }\underset{i>j\in I}{\sum }\alpha \left(\left|i-j\right|\right)f\left(r_{ij}\right)+\underset{i>j}{\sum }\alpha \left(T_{i},T_{j}\right)f\left(r_{ij}\right), \end{array}$$ (3)

where *I* represents indices over chromosomes and *i* and *j* represent individual genomic loci, respectively. *U*(***r***_*I*_) is the generic potential as that in Eq. (2) to account for polymer topology. The second term corresponds to the ideal chromosome potential that depends only on the sequence separation but not on compartment types. It accounts for the interactions within the same chromosome and could arise from protein-mediated contacts^38,116–118,123,127,145,146^ and spherical confinement.^93,147^ Compartment type (*T*_*i*_ ∈ {*A*, *B*}) specific interactions are included in the last term. The energy function, *U*_Genome_(***r***), can again be derived by maximizing the excess entropy while enforcing experimental constraints that measure the average contact probabilities at various sequence separations and between different compartment types.^144^ Parameters in the energy function, *α*(|*i* − *j*|) and *α*(*T*_*i*_, *T*_*j*_), can be uniquely determined with the iterative optimization algorithm mentioned in the Sec. II B 1 b.

As shown in Fig. 4 B_1_, this model succeeds in recapitulating the microphase separation between the two compartments and the checkerboard pattern seen in Hi-C contact maps. However, a close examination of the simulated genome conformations suggests that heterochromatin (*B* compartments) is scattered over the entire nucleus rather than localizing to the nuclear periphery, as shown in EM images.^36^ Notably, the unique modeling strategy employed here ensures that the inconsistency between simulation and experiment cannot be resolved by fine-tuning the model parameters, the values of which were inferred from experimental data optimally. The hypotheses introduced in designing the model must be incorrect or incomplete. Therefore, cross-validating simulated structures with imaging experiments offers a feedback mechanism to falsify hypotheses of genome organization mechanisms.

After revisiting the model hypotheses, we found that decoupling the intra- and inter-chromosome interactions is necessary and sufficient for reproducing the peripheral localization of *B* compartments (Fig. 4 B_2_). This decoupling indicates the presence of different mechanisms that drive phase separation at various scales. Indeed, it is known that protein-mediated interactions dominate at intra-chromosome contacts.^148^ On the other hand, various nuclear landmarks, including lamina and speckles,^149,150^ could contribute to the aggregation of various chromosomes. These distinct mechanisms necessitate the use of two sets of independent parameters for intra- and inter-chromosome interactions.

We further found that a third compartment type that corresponds to the central region of chromosomes, or centromeres, is needed to reproduce the radial position of individual chromosomes (Fig. 4 B_3_). Including this type helps capture centromere clustering that has been seen in prior studies.^151^ We applied the modeling strategy to study the global rearrangement of genome organization upon tumorigenesis^152^ and observed a remarkable change in genome organization upon tumorigenesis. Unlike their interior localization in normal samples, *A* compartments in tumors are scattered across the entire nucleus. This observation was later confirmed by electron microscopy.

Most existing computer models do not explicitly include nuclear landmarks such as lamina, nucleoli, and speckles, although studies that account for them are emerging.^85,86,153–156^ Modeling the landmarks and the genome together could potentially circumvent the need for an artificial distinction between intra- and inter-chromosome interactions as in the current model. A careful investigation of the coupling between the genome and nuclear lamina may provide insight into the mechanical properties of the nucleus and the impact of mechanical forces on gene transcription.^157,158^ Such models could also reveal the role of chromatin network in both the kinetics and thermodynamics of phase separation that drives nucleolus and speckle formation.^156,159,160^

#### 2. Predicting chromosome structures from epigenetic marks

**FIG. 5.**
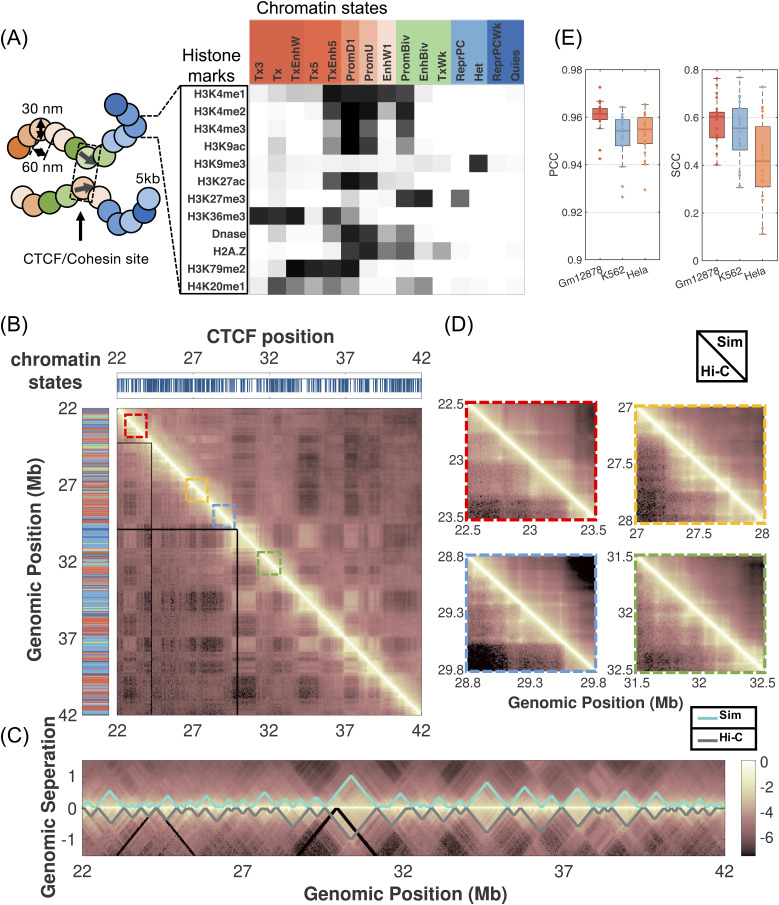
Predicting genome organization with a chromatin-state based polymer model. (a) Overview of the key elements of the computational model. The chromatin is modeled as a string of beads, each assigned with a chromatin state based on the corresponding combinatorial pattern of histone marks. Genomic regions bound by CTCF molecules are also identified to model CTCF mediated loop formation. The polymer model succeeds in quantitatively reproducing compartments (b), TADs (c), and chromatin loops (d) for chromosome 1 from GM12878 cells. (e) The polymer model is transferable across chromosomes and cell types as evidenced by the high correlation between simulated and experimental contact maps measured by Pearson correlation coefficients (PCC—left panel) and stratum-adjusted correlation coefficient (SCC—right panel).

In addition to the whole-genome organization, we applied the data-driven mechanistic modeling strategy to study the structure of single chromosomes at 5kb resolution.^161^ The model’s higher resolution allows detailed characterization of fine-scale structures, including TADs and loops, which play essential roles in gene regulation. Chromosomes can again be viewed as block copolymers, but now the monomer types go beyond the *A*/*B* compartments used in whole-genome modeling. Instead, we represented a chromosome as a sequence of chromatin states that were defined as unique combinatorial patterns of histone modifications [see Fig. 5(a)]. Chromatin states and *A*/*B* compartments are related in many ways. In particular, the states marked with histone modifications H3K4me3 and H3K27ac often correspond to *A* compartments, while those marked with H3K9me3 or H3K27me3 overlap with *B* compartments. Chromatin states provide a more nuanced view of the variety of chromatin types and a molecular interpretation for the abstract compartments derived from Hi-C data. We note that the use of multiple representations for polymer modeling is not uncommon in the language of protein folding: while two types of amino acids, hydrophobic and hydrophilic, are sufficient to understand the collapse of protein molecules, the chemical specificity of individual amino acids becomes important for predicting high-resolution structures.

We followed the same procedure as whole-genome modeling to define an energy function and derive parameters by enforcing simulated ensemble averages to match average experimental contact probabilities. Details of the model definition and parameterization can be found in Ref. 161. Computer simulations of this model provided a high-resolution structural characterization of chromatin loops, TADs, and compartments. They succeeded in quantitatively reproducing contact probabilities and power-law scaling of 3D contacts, as measured in Hi-C and super-resolution imaging experiments [Figs. 5(b)–5(d)]. In particular, we found that for chromosome 1 of GM12878 cells, the correlation between simulated and experimental contact map exceeds 0.96. The stratum-adjusted correlation coefficient (SCC),^162^ which considers the distance-dependence effect of contact maps by stratifying them according to the genomic distance, is 0.7. This value is comparable to that between the experimental replicates. We further examined the agreement between simulated and experimental contact maps using multiple feature-specific metrics. We found that our model reproduces over 74% of the CTCF-mediated chromatin loops, correctly identifies ∼80% of the TAD boundaries,^163^ and captures over 57% of the significant enhancer–promoter contacts.

In addition to producing high-resolution chromosome structures, the chromatin-state based model further enables *de novo* prediction. For example, it can be applied to any chromosome, as long as the corresponding sequence of chromatin state is provided as input. Unlike *A*/*B* compartments that can only be derived from Hi-C data, defining chromatin states only requires histone modifications, and no Hi-C data are needed for structure prediction. We performed additional simulations for chromosomes from GM12878, K562, and HeLa cells, which were not used in model parameterization. We found that the simulated Hi-C data are in good agreement with experimental results as well.

Several other groups have carried out predictive modeling of genome organization using histone modifications with great success as well.^139,164,165^ For example, Di Pierro *et al.* simulated chromosome structures at the 50kb resolution using chromatin types defined with histone modification profiles and trained to mimic genomic compartments.^135^ MacPherson *et al.* modeled chromosome 16 at the single nucleosome resolution.^164^ Using the H3K9me3 pattern derived from ChIP-seq signals, they reproduced the phase separation of euchromatin and heterochromatin seen in Hi-C contact maps. The accuracy of these epigenetic-mark-based models can be further improved. In particular, many protein molecules are known to mediate genomic contacts by directly engaging with the underlying nucleotides. A representation based purely on histone modifications is not sufficient to capture such DNA sequence-specific effects. Encouraging progress is being made at predicting these interactions from the DNA sequence using convolution neural networks.^166–168^ Incorporating these studies into the polymer model could provide a more accurate description of genome organization.

## III. STRUCTURE FOR A STRING OF NUCLEOSOMES

The whole-genome modeling effort and many other studies^169,170^ firmly establish the importance of histone modifications in genome organization. Predicting chromosome structures from chromatin states provides further evidence for the sequence–structure relationship of the genome. This relationship suggests that tinkering the post-translational modifications could serve as effective means to alter genome organization and gene expression. Epigenome engineering that aims to modify such epigenetic marks rather than the genetic code has indeed been pursued as a complementary and less invasive approach to genome engineering.^171,172^

To effectively alter chromatin types for epigenome engineering and ensure robust and long-lasting changes in histone modifications, it is essential to study mechanisms that dictate their stability. Histone modifications are subject to constant perturbations from addition and removal enzymes inside the nucleus.^173,174^ The chromatin structure has been implicated in mediating the spread and maintenance of histone marks by these enzymes.^136,175–180^ Close contacts can facilitate the transfer of enzymes from modified nucleosomes to unmodified ones, introducing cooperativity among the modifications.^181^ Therefore, studying the stability of histone modifications necessitates a detailed characterization of chromatin organization at the resolution of tens of nucleosomes.

### A. The 30 nm fiber

**FIG. 6.**
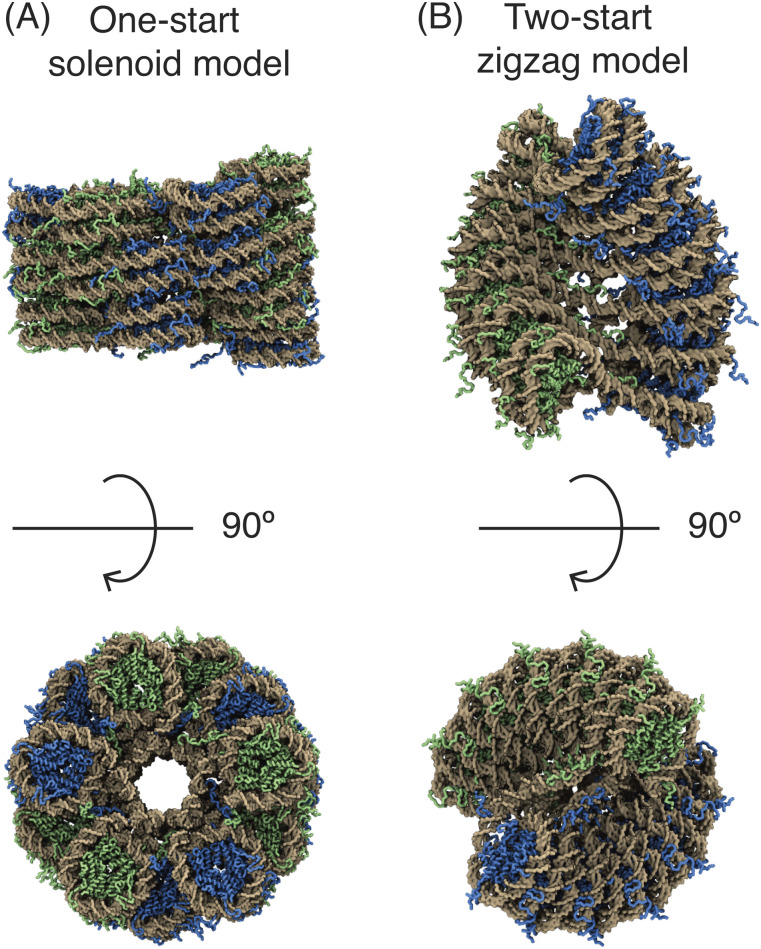
Illustration of the two structural models proposed for chromatin fiber. A total of 24 nucleosomes are shown in panel (a) and 12 nucleosomes in panel (b). Histone proteins from the odd and even nucleosomes are shown in blue and green, respectively. The DNA molecule is indicated in gold.

Packaging the 2-m long human genome inside a nucleus of 10 *μ*m in diameter is a daunting task. Nucleosomes and TADs fold the genome at 200 bp and 1Mb, respectively. Additional compaction occurs to bridge the two rather different length scales. For example, Finch and Klug hypothesized that the 10 nm fiber as a string of nucleosomes could coil into a solenoid to form a 30 nm fiber.^182^ As shown in Fig. 6(a), nucleosomes are packed face-to-face around a central axis in the solenoid model, and each helical turn encloses six or seven nucleosomes. Additionally, Woodcock and co-workers introduced the two-start model based on the electron tomography data that exhibited two nucleosome-wide ribbons.^183,184^ The two-start model predicts a zigzagging of two nucleosomes that coil into a helical conformation of roughly 30 nm [Fig. 6(b)]. Other fibril models that differ in the nucleosome path have also been proposed.^185^ Support of these different structural models mostly come from EM images of the chromatin material extracted from the nucleus under harsh conditions. The use of *in vitro* reconstituted nucleosome arrays helped in removing sample heterogeneity in nucleosome spacing and made possible the determination of high-resolution structures with cryo-EM. These studies further support the presence of regular fibril conformations,^186^ and the more recent atomic structure favors the two-start zigzag model.^187^

However, the quest for 30 nm fibers inside the nucleus has often led to disappointing conclusions. Many research groups have failed to confirmed its presence employing a wide range of techniques, including cryo-EM,^188^ electron spectroscopic imaging,^189^ small-angle x-ray scattering,^190,191^ super-resolution imaging,^192^ Hi-C,^122^ and ChromEM tomography.^193^ It is becoming increasingly clear that chromatin organization at the kilobase scale is sensitive to a variety of factors,^32,194^ including salt concentration, nucleosome spacing, and interaction with non-histone proteins. A systematic characterization of these various factors will be essential for revealing key physicochemical interactions that drive chromatin folding and reconcile the seemingly contradictory experimental observations. However, it has proved challenging due to difficulties in precise chromatin engineering and assembly.

### B. Mesoscopic modeling of chromatin fiber

Computational modeling offers an alternative and promising approach for studying chromatin organization at the kilobase scale. Compared to whole-genome modeling, the problem here is more straightforward, at least conceptually, since the system is well defined. All the components are known, and one can, in principle, carry out all-atom simulations for *de novo* structure prediction.^195,196^ Materese *et al.* have shown that such simulations could provide insight into the hydration and electrostatic environments near the nucleosome^197^ and the dependence of nucleosome elastic properties on histone variants.^198^ Shaytan *et al.* revealed a conformational coupling between histone tails and nucleosomal DNA, with histone tail binding promoting DNA bulging and twisting.^199^ The Collepardo-Guevara *et al.* characterized the role of histone modifications on chromatin compaction and the importance of linker histone H1 on chromatin hierarchical looping.^200,201^ Winogradoff and Aksimentiev further reported direct observation of spontaneous DNA unwrapping and characterized the role of CG content in such motions.^202^ Recently, the Wereszczynski laboratory applied atomistic simulations to study the impact of linker histones on the structure of an octa-nucleosome array.^203^ However, a minimum system for a string of nucleosomes with explicit solvation can easily exceed several millions of atoms,^204^ making it challenging to carry out such simulations over long timescales and quantify the stability of various chromatin conformations. Over the years, many coarse-grained models that differ in structural details and energetic terms have been introduced to study chromatin organization.

**FIG. 7.**
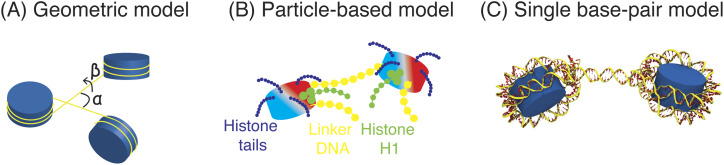
Illustration of the three different types of mesoscopic chromatin models that differ in the representation and energetic contributions. (a) In geometric models, the energetics of the chromatin fiber is fully specified by two angles that correspond to the DNA entry–exit angle of individual nucleosomes (*α*) and the relative rotational angle between two connecting nucleosomes (*β*). (b) Particle-based models allow for more accurate treatment of the flexibility of linker DNA and histone tails and histone H1. Inter-nucleosome interactions can be introduced to account for contributions from globular domains of histone proteins. (c) Models with DNA molecules at a single base-pair resolution have been introduced to characterize the bending and twisting of linker DNA with greater details.

The two-angle model introduced by Woodcock *et al.* has been instrumental in providing a conceptual framework for studying the chromatin organization^205^ [see Fig. 7(a)]. The angles correspond to the DNA entry–exit angle of individual nucleosomes (*α*) and the relative rotational angle between two connecting nucleosomes (*β*). Varying the two angles can give rise to a wide variety of fibril conformations with different arrangements of nucleosomes,^206,207^ including the two popular models proposed for the 30-nm fiber: one-start solenoid and two-start zigzag. The dependence of chromatin conformation on histone modifications, histone H1 binding, and linker length can be understood from their impact on the angles.^186,208^

The two angle model’s success encouraged the development of more refined models that go beyond geometric arguments. For example, accounting for the elasticity of linker DNAs and interactions between nucleosomes^209^ allowed Katritch *et al.* to reproduce the force-extension characteristics of chromatin fiber measured by single-molecule pulling experiments.^210^ Wedemann *et al.* further incorporated the electrostatic interactions between linker DNAs to quantify the stability and persistence length of the chromatin fiber and characterize the transition between different fiber structures induced by the binding of linker histone H1.^211,212^ Schlick and co-workers introduced a chromatin model that employs particle-based representations for histone tails, linker DNA, and linker histones and accounts for the charge distribution of the nucleosome core particle^213,214^ [Fig. 7(b)]. This model was shown to reproduce a variety of experimental observations, including the dependence of chromatin conformation on salt concentration^213^ and histone modifications,^200,215^ and the compaction of chromatin fiber upon the addition of divalent ions and linker histones.^216^ Lequieu *et al.* developed the 1CPN model that connects nucleosome core particles with flexible linker DNA.^217^ Notably, inter-nucleosome interactions were not computed from the mesoscopic representation but parameterized using free energy calculations carried out with a near-atomistic model. Such a multi-scale strategy could lead to a systematic improvement of model accuracy without sacrificing computational efficiency.

The strong dependence of chromatin organization on the linker DNA length further inspired the development of models with a single-base resolution for the DNA [Fig. 7(c)]. For example, Norouzi and Zhurkin used a knowledge-based potential to evaluate the elastic energy of the linker DNA deformations.^218^ Together with electrostatic interactions and specific internucleosomal interactions, the resulting model predicted the presence of different topoisomers that are favored by chromatin with linker DNA of 10n and 10n + 5 bp in length. The model was further applied to interpret experimental chromatin force-extension curves and dissect the contributions of nucleosome unwrapping and unstacking.^219^ de Jong *et al.*^220^ applied a rigid-base-pair DNA model to describe un- and re-wrapping of nucleosomal DNA and bending and twisting of linker DNA. Monte Carlo simulations with this model support the stability of different fiber models, two-start and one-start, for chromatin with linker lengths of 20 and 50 bp. A similar model that accounts for the bending and twisting of linker DNA was employed by Koslover *et al.*^221^ to study the dependence of the nucleosome path in chromatin fibers on the linker DNA length.^186^ The model predicts a number of energetically comparable configurations with different nucleosome–nucleosome interaction patterns, indicating a potential role for kinetic trapping in chromatin fiber formation.

### C. Perspective: Near atomistic chromatin modeling

The prior studies have greatly enriched our understanding of the various conformations isolated chromatin can adopt. To further characterize chromatin organization *in situ* and reconcile the difficulty for detecting the 30 nm fiber inside the nucleus, one must study the complex interaction between chromatin and the nucleus environment.^22,200,193^ Increasing evidence suggests that intrinsically disordered proteins (IDPs) that interact strongly with chromatin can spontaneously form liquid droplets.^222–228^ Collective interactions with these proteins can drive the chromatin into highly dynamic conformations that differ significantly from rigid fibril structures.^229,230^ Therefore, accounting for protein chromatin interactions will be essential to model chromatin organization *in situ* but can be challenging with the existing mesoscopic models.

Implicit solvent near-atomistic models offer a promising approach for a detailed characterization of protein–protein and protein–DNA interactions at a single base and residue level. They allow a rigorous treatment of electrostatic, van der Waals, and other interactions between particles based on physical chemistry. Several models have been introduced for the DNA.^231^ For example, de Pablo and co-workers developed a DNA model that uses three sites per nucleotide (3SPN).^232–234^ Parameters of the model were chosen to reproduce the experimentally measured free energies of base stacking and hybridization. The model was shown to reproduce many properties of DNA molecules accurately, including the persistence length of both single and double-stranded DNA under physiological conditions, the dependence of persistence length on salt concentration and DNA sequence, DNA melting temperatures, and hybridization rate constants.

Integrating 3SPN DNA with similarly coarse-grained protein models has enabled both quantitative and qualitative investigations of chromatin organization. The de Pablo group modeled histone proteins at one bead per amino acid resolution with the atomistic-interaction based coarse-grained protein model (AICG).^235^ Interactions among amino acids were parameterized from energies and dynamics of all-atom models via a multiscale protocol. They applied the combined protein–DNA model to reveal that sequence-specific histone binding affinity of the DNA molecule is encoded in their shape.^234^ The group further uncovered the coupled role of the DNA-sequence, histone modifications, and chromatin remodelers in positioning nucleosomes^236^ and the dependence of nucleosome unwinding barrier on applied tension.^237^ Finally, they showed that the model succeeded in reproducing the binding strength between a pair of nucleosomes measured in DNA origami-based force spectrometer experiments.^238,239^ Using a closely related protein–DNA model, the Kenzaki and Takada studied the dynamics of DNA unwinding,^240^ transcription-factor binding to nucleosomes,^241–243^ structures of di- and tri-nucleosomes,^244^ and nucleosome sliding/remodeling and twist propagation.^245,246^ These studies highlight the usefulness of near-atomistic models in uncovering detailed mechanisms that are otherwise challenging to extract from either experimental studies or mesoscopic models.

To model a nucleosome at a high resolution, we combined 3SPN DNA with the associative memory, water-mediated, structure, and energy model (AWSEM) for protein introduced by Davtyan *et al.*^247^ Each amino acid was represented with three beads for the *C*_*α*_, *C*_*β*_, and *O* sites. Interactions among amino acids were optimized following the energy landscape theory prescription to maximize the ratio of folding temperature over glass transition temperature and sculpt a funneled folding landscape for a set of training proteins.^248–250^ Similar to the studies of the de Pablo and the Takada groups, protein–DNA interactions were modeled with the Debye–Hückel theory to account for water’s dielectric properties and the screened electrostatic interaction. Because of the lack of base-specific hydrogen bonding between histone proteins and the nucleosomal DNA as seen in the crystal structure,^251^ electrostatic contributions are expected to dominate the interactions between them.

**FIG. 8.**
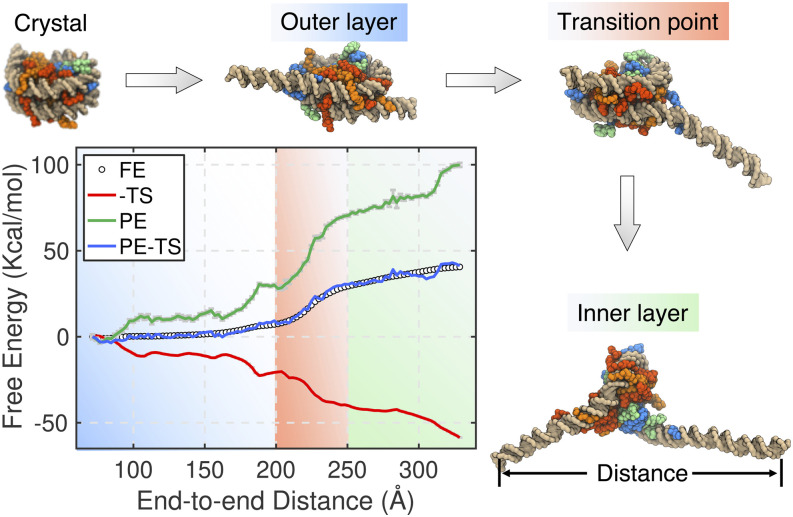
Thermodynamics of nucleosome unwinding. The free energy (FE) profile (white dots) as a function of the DNA end-to-end distance supports a three-stage scenario for DNA unwinding. The first stage (blue) corresponds to the unwinding of the outer layer. In the second stage (orange), no significant DNA unwinding occurs, but free energy rises sharply. Finally, the inner layer begins to unwind at a modest free energy cost in the third stage (green). Example nucleosome configurations at different stages are provided on the side, with the DNA indicated in gold and histone proteins indicated in orange, red, blue, and green. The free energy barrier in the transition region is mostly dominated by energetic contributions (*PE*, green line), which are compensated by an increase in entropy (−*TS*, red line) from the freed histone tails.

Using the near-atomistic model for protein and DNA molecules, we determined the free energy landscape for nucleosome unwinding.^252,253^ Our study revealed a sizable energetic barrier that decouples the unwinding of the 147 bp long DNA molecule into two separate processes (Fig. 8). This barrier height is in quantitative agreement with the value determined by Brower-Toland *et al.* using single-molecule force spectroscopy^254^ and by the Lequieu *et al.* using computer simulations of a closely related near-atomistic model.^237^ We note that the mechanistic origin of the energetic barrier has been the focus of several theoretical studies.^255–257^ Via a rigorous thermodynamic analysis, we found that the barrier mainly arises from a delayed loss of contacts between disordered histone tails and the DNA. This delay is caused by the dynamical relocation of disordered tails to preserve their DNA contacts. Surprisingly, the energetic penalty is largely offset by an entropic contribution from these newly freed tails. The enthalpy–entropy compensation mechanism provided a fresh perspective on nucleosome stability and highlighted the importance of studying chromatin organization at a high resolution.

We further studied the folding of the basic unit of the chromatin fiber, the tetra-nucleosome.^258^ The *C*_*α*_ protein model that represents each amino acid with one bead^259,260^ was used to reduce the computational cost. While lacking the sophisticated energy functions used in AWSEM, it suffices to capture the histone octamer’s conformational fluctuation around the crystal structure. Direct simulations of tetra-nucleosome folding from extended configurations to collapsed structures are not feasible. Folding will be hindered by slow dynamics that arise from the break and formation of strong, non-specific electrostatic contacts. An advanced sampling technique^261^ that combines metadynamics^262^ with temperature accelerated molecular dynamics simulations^263,264^ enabled efficient conformational sampling and produced various tetra-nucleosome configurations.

**FIG. 9.**
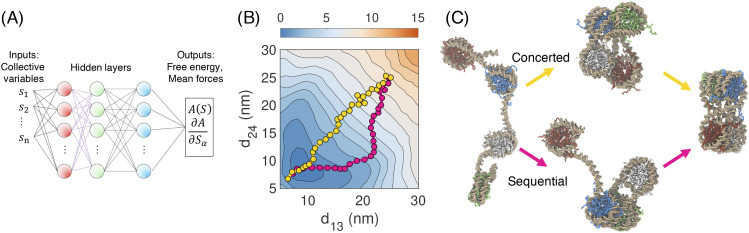
Stability and folding pathways of the tetra-nucleosome. (a) Illustration of the neural network approach for parameterizing high-dimensional free energy surfaces from mean forces. The neural network takes the six internucleosomal distances (*S*_1_, *S*_2_, …, *S*_*n*_) as an input to compute the corresponding free energy [*A*(***S***)] and mean forces ($\frac{\partial A}{\partial S_{\alpha }}$). (b) Projection of the six-dimensional free energy profile to the distance between 1 and 3 (*d*_13_) and 2 and 4 (*d*_24_) nucleosomes. The sequential (pink) and concerted (yellow) pathway for tetra-nucleosome folding are shown on top of the free energy profile with energy unit kcal/mol. (c) Example tetra-nucleosome configurations along the two folding pathways. The DNA molecule is shown in gold, and the histone octamers are shown in green, white, blue, and red.

To further evaluate the stability of the simulated tetra-nucleosome configurations, we computed the free energy surface as a function of the six inter-nucleosomal distances (Fig. 9). The use of a large number of collective variables is needed to resolve the different conformations but poses challenges to traditional methods such as umbrella sampling. Instead, we determined the surface with a neural network approach by integrating mean forces collected at a series of preselected centers.^265^ The free energy surface supports the global stability of the stacked, zigzag configuration resolved in x-ray crystallography^266^ and cryo-EM^187^ as part of the 30 nm fiber. We determined the most probable folding pathways, or the minimum free energy paths, from open configurations to the zigzag structure using the finite-temperature string method.^267^ Notably, the pathways go through intermediate configurations that resemble chromatin configurations observed *in situ*.^268,269^ Our study suggests that chromatin configurations observed *in situ* are closely related to the *in vitro* fibril structures and may form as a result of local excitations or unfolding from the global minimum.

The folding intermediates bear comparable stability as the zigzag structure and can be further stabilized by configurational entropy, histone modifications, and variation in the secondary structure of the histone tail. Therefore, chromatin organization is sensitive to both thermal and chemical perturbations. Given the complexity of the nucleus environment, it is perhaps not too surprising to frequently observe the folding intermediates. Chromatin indeed favors more disordered configurations in which proteins mediate contact between non-neighboring nucleosomes,^181^ when the binding of the chromatin regulator, Polycomb Repressor Complex 2 (PRC2), was taken into account.

#### 1. Study the role of phase separation in chromatin organization with MOFF

The techniques used for tetra-nucleosome folding can be generalized to longer chromatin to evaluate the stability of various fibril structures and the dependence of their stability on the linker DNA length. However, to study the impact of chromatin regulators, a more accurate protein force field is needed. Many chromatin regulators contain disordered regions, and recent evidence suggests that the phase separation of these proteins^270–273^ drives changes in the chromatin structure.^222,223,234^ We found that the existing coarse-grained force fields, which were often parameterized to fold globular proteins and predict protein structures,^247,274,275^ tend to produce overly compact conformations for IDPs. As such, there is widespread interest in developing force fields specifically suited for IDPs.^276–278^ Despite the advances, algorithms, which can drive systematic improvements in force field accuracy and ultimately reconcile differences between folded and unfolded models, remain scarce, although improvements have been made in the fully atomistic case.^279–283^

The maximum entropy optimization algorithm introduced in whole-genome modeling is well suited to improve the existing force fields for IDPs. For example, our group and others have incorporated low-resolution experimental data from small-angle x-ray scattering (SAXS) into computer models to refine structure prediction.^284–286^ However, an obvious drawback of this approach is that it cannot be applied to proteins for which no experimental data are available.

**FIG. 10.**
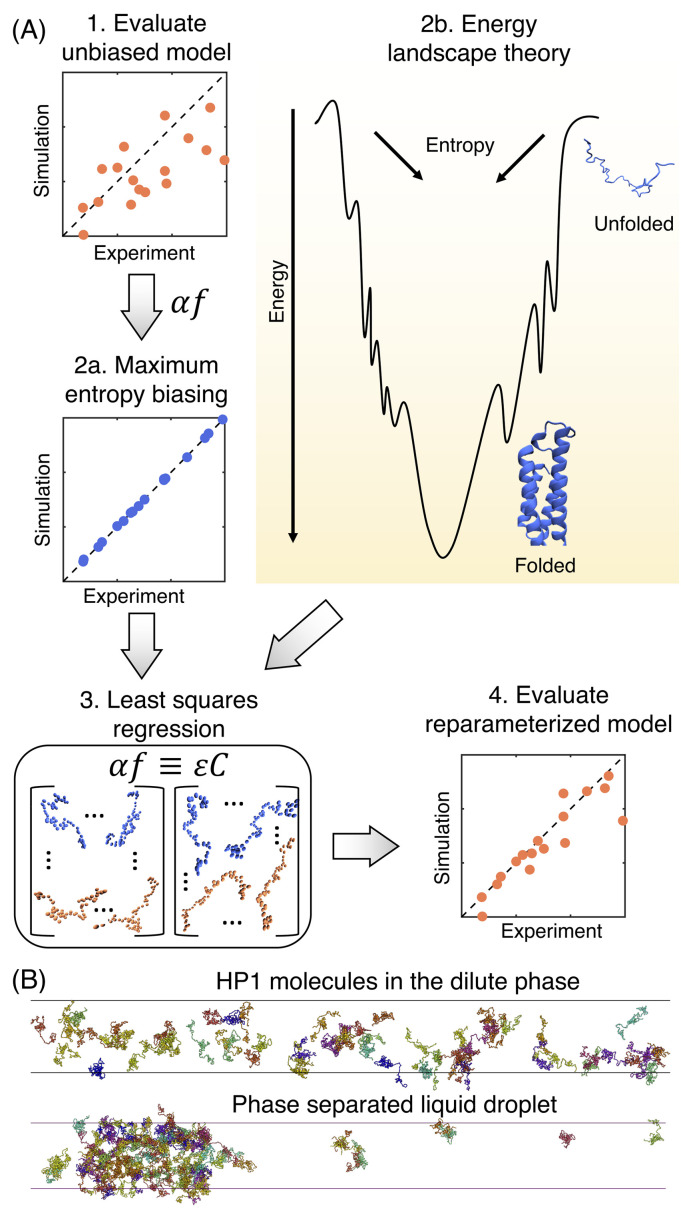
Coarse-grained protein force field, MOFF, enables large scale simulation of phase separation. (a) Illustration of the maximum entropy optimization algorithm for protein force field parameterization. The algorithm reparameterizes biasing energies (*αf*) determined from maximum entropy optimization with a weighted linear combination of contacts (*ɛC*). When solving the reparameterization algorithm with least squares regression, additional constraints can be included for globular proteins to ensure that the native conformations have the lowest energy (step 2b in yellow). (b) Example configurations of HP1 molecules in the dilute and condensed phase.

We developed another algorithm to parameterize a transferable force field for IDPs, which we term maximum entropy optimized force field for IDPs or MOFF-IDP.^287^ The algorithm consists of three steps, as outlined in Fig. 10(a). First, simulations are performed for a set of IDPs with an initial force field to *evaluate the unbiased model*. Then, *maximum entropy biasing* is used to determine the least biased correction energies (*αf*) that reproduce protein radii of gyration derived from SAXS experiments. Third, *least squares regression* parameterizes the protein-specific biasing energies with pairwise amino acid potentials (*ɛC*) to ensure transferability. These three steps can be repeated to improve the force field accuracy further. The resulting force field, MOFF-IDP, performed well for *de novo* prediction of IDP structural ensembles. For example, it captured the structural rearrangement in the epidermal growth factor receptor C-terminus domain upon phosphorylation.^288^

Just like how the force fields optimized for protein folding and structural prediction cannot be applied to IDPs, most IDP force fields, including MOFF-IDP, are not transferable to globular proteins either. Since both globular proteins and IDPs consist of the same 20 amino acids, it is plausible and desirable to have a consistent force field that describes both types of proteins equally. The maximum entropy optimization algorithm can be readily extended to include folded proteins to improve the force field transferability. Furthermore, it could be combined with other ideas introduced for force field optimization.

Recently, we extended the maximum entropy optimization algorithm with ideas from protein folding studies.^289^ For example, energy landscape theory, which succeeded in providing a conceptual framework for studying protein folding kinetics and thermodynamics, requires the protein force field to be funneled toward the native configuration for reliable and efficient folding of globular proteins.^256^ Therefore, we added globular proteins to the training set for the first two steps of the algorithm. We further enforced additional constraints for folded proteins when solving the reparameterization equations to require the energy of the native structures to be lower than that of the misfolded ones for these proteins [see step 2b of Fig. 10(a)]. The reparameterized force field (MOFF) succeeded in predicting the size of both globular and disordered proteins with consistent accuracy.

We have begun to utilize MOFF to study heterochromatin protein 1 (HP1), an essential chromatin regulator known to phase separate. Our results help explain the experimental observations of homolog specific phase separation [Fig. 10(b)].^222,290^ When combined with the DNA model mentioned in Sec. III C, MOFF could help address the role of phase-separating proteins in chromatin organization with near-atomistic details.

## IV. PERSPECTIVE: CHROMOSOME DYNAMICS

Hi-C, imaging, and related methods have provided a comprehensive characterization of genome organization. To connect the structure with function, however, a detailed understanding of chromosome dynamics is required. A prominent example is the eukaryotic gene activation facilitated by the contact formation between enhancers and promoters. Whether these contacts are transient, as in the hit-and-run mechanism,^291^ or persistent^292^ remains controversial. Distinguishing the two mechanisms have significant implications on genome engineering to control gene expression. Chromosome movement is crucial for the sequence-based homology searches during double-strand break repair,^293^ gene rearrangements in antigen receptor repertoire establishment,^294^ and telomere length regulation^295^ as well. Therefore, a full understanding of the functional role of 3D genome organization can only be achieved by accounting for its dynamical component.

Live-cell imaging has provided great insight into chromatin dynamics.^15,296^ When the positions of individual loci were tracked over time, a subdiffusive, visco-elastic motion was observed.^294,297,298^ In contrast to regular diffusion, the mean squared displacement of genomic loci scales with time as *t*^*α*^ with *α* < 1. Chromatin dynamics was further found to be highly heterogeneous and is sensitive to a variety of factors, including the nuclear localization,^299,300^ the length of the loci,^301,302^ and the concentration of lamin A/C protein.^303^ Mapping chromatin dynamics across the entire nucleus using Green fluorescent protein (GFP)-tagged histone H2B^304^ or fluorescent-labeled DNA^305^ uncovered surprisingly long-range correlation among genomic loci over the micrometer scale. This correlated motion is at least partially driven by ATP or transcription.

Experimental findings have inspired numerous theoretical studies.^306,307^ Much progress has been made to understand the origin of anomalous diffusion in a crowded environment. The elastic interaction between a locus and its neighboring consecutive segments because of the polymer nature of the genome alone could give rise to sub-diffusive behavior at intermediate timescales.^308^ The folded organization of chromosomes can lead to quantitative changes in the scaling exponent.^309,310^ Passive interactions between genomic loci and the nuclear compartments could further restrict their diffusivity via the so-called continuous-time random walk (CTRW).^311^ Finally, a particle moving through a viscoelastic environment will undergo subdiffusive motion over a range of time scales due to elastic stresses within the medium.^294,312–314^ This environment may, in fact, arise from the chromatin itself, which has been shown to exhibit glassy^131^ and gel-like property.^315–317^

While theoretical studies have been very successful at deriving general principles, they often have to introduce significant assumptions regarding the biological complexity of the underlying system to make the problem analytically solvable. Consequently, these studies face challenges in explaining the heterogeneity of single-locus dynamics. In addition, generalizing them to the whole genome is non-trivial. The correlated motion among loci is naturally a more complex problem. Hydrodynamics, motors, nuclear compartments, and phase separation all could potentially contribute to such a correlation. Computer simulations that treat the different factors on equal footing could quantify their contributions to chromosome dynamics. The detailed microscopic mechanism arising from numerical simulations could inspire more quantitative theories on chromatin dynamics.

The whole-genome model introduced in the Sec. II will be valuable for studying chromosome dynamics. For example, it will be straightforward to characterize the subdiffusive behavior of telomeres and understand the mechanistic origin that gives rise to the heterogeneity among different loci. In addition to the behavior of individual loci, the dynamical correlation among loci can also be characterized by computing the spatial–temporal correlation defined as

$$C^{\Delta t}\left(r\right)={\left\langle \frac{\sum_{i>j}\left[\Delta r_{i}\left(t;\Delta t\right)\cdot \Delta r_{j}\left(t;\Delta t\right)\right]\delta \left(r_{i,j}\left(t\right)-r\right)}{\sum_{i>j}\delta \left(r_{i,j}\left(t\right)-r\right)}\right\rangle }_{t},$$ (4)

which quantifies the displacement correlations between loci separated by a distance *r* over the time interval Δ*t*. The angular brackets represent averaging over time. Prior simulation studies have shown that correlated motions are present within individual chromosomes due to chain organization.^318,319^ The whole-genome model offers a unique opportunity to examine the correlation over scales beyond chromosome territories.^304^

A complete understanding of chromosome dynamics cannot be achieved without accounting for the role of ATP-driven remodeling enzymes. These enzymes can affect the dynamics of single loci^320–322^ and the large scale correlated motions. The whole-genome model can be modified straightforwardly to study non-equilibrium dynamics, at least approximately, by introducing colored noise to the equation of motion.^323^ In addition to numerical simulations, it is crucial to develop analytical approaches that help conceptualize the impact of non-equilibrium motors on the chromatin structure and dynamics. Wang and Wolynes introduced a perturbation theory to map the non-equilibrium steady state in terms of an effective temperature via a systematic expansion of the many-body master equation.^139,324^ This approach is appealing as it could open up the door of applying equilibrium statistical mechanical theories for non-equilibrium systems. We followed similar ideas^140,325^ to show that the effect of ATP-driven chromatin remodeling enzymes on nucleosome positioning could be well approximated by effective equilibrium models with rescaled temperatures and interactions. Numerical simulations support the theory’s accuracy in predicting both kinetic and steady-state quantities, including the effective temperature and the radial distribution function, in biologically relevant regimes. Generalizing these studies could complement numerical simulations to understand the role of molecular motors in genome organization.

It is worth noting that the force field derived from Hi-C data only provides an effective energy landscape, which is not guaranteed to reproduce dynamical measurements. The maximum caliber method,^326,327^ which is a generalization of the maximum entropy principle for dynamical trajectories, could be used to incorporate dynamical information^328,329^ and improve the dynamical properties of the whole-genome model.

## AUTHORS’ CONTRIBUTIONS

X.L. and Y.Q. contributed equally to this work.

## Data Availability

The data that support the findings of this study are available from the corresponding author upon reasonable request.
